# Mitigating Recombination Losses in CZTSSe Solar Cells via Interface Engineering: A Comprehensive Review

**DOI:** 10.3390/molecules31132286

**Published:** 2026-06-30

**Authors:** Xuanyu Liu, Yuqing Xiao, Yuhong Jiang, Hanxi Gong, Yiming Xia, Dandan Wang, Bin Yao, Jinghai Yang, Yong Zhang

**Affiliations:** 1 Key Laboratory of Functional Materials Physics and Chemistry of the Ministry of Education, Jilin Normal University, Changchun 130103, China; 2 College of Physics, Jilin University, Changchun 130012, China

**Keywords:** Cu_2_ZnSn(S,Se)_4_ (CZTSSe), interface engineering, thin-film solar cells, V_oc_ deficit, defect passivation

## Abstract

As an emerging photovoltaic technology, Cu_2_ZnSn(S,Se)_4_ (CZTSSe) thin-film solar cells are regarded as a viable, cost-effective alternative to satisfy future demand for green energy. This promise is attributed to their tunable bandgap (1.0~1.5 eV), high absorption coefficient (>10^4^ cm^−1^), and environmentally friendly composition. Currently, the record power conversion efficiency (PCE) of CZTSSe devices has reached 16.6%, approaching commercial levels. However, this value remains significantly lower than its theoretical limit of 32.8% and the 23.6% achieved by the homologous CIGS technology, indicating immense potential for performance enhancement. The severe open-circuit voltage deficit (E_g_/q-V_oc_) remains a critical factor preventing CZTSSe solar cells from reaching their expected efficiency. This issue is primarily associated with band misalignment and deep-level defects at the interfaces. At present, interface engineering has been demonstrated to be an effective strategy to significantly improve the performance of CZTSSe thin-film solar cells. Herein, we review the development process of CZTSSe photovoltaics, systematically discuss existing interface-related issues and comprehensively summarize recent strategies in interface engineering. Finally, to further elucidate the intrinsic mechanisms and facilitate the development of high-efficiency devices, future research directions and perspectives regarding interface engineering are proposed.

## 1. Introduction

Since the 20th century, global energy demand has grown substantially along with rapid industrialization, exposing the limitations of a long-term reliance on fossil fuels. Therefore, the global energy transition has entered a complex stage [1]. In this context, solar photovoltaic (PV) technology, which is clean, renewable, ubiquitous, and environmentally friendly, is increasingly becoming one of the pillars of the new energy infrastructure. Currently, the development of high-efficiency, low-cost next-generation solar cells serves as the critical foundation for achieving the widespread application of solar photovoltaic technology [2,3,4,5].

It is reported that single-junction crystalline silicon (c-Si) solar cells have a record PCE of 27.8% [6,7]. Nevertheless, their inherent limitations, such as high manufacturing costs and a relatively low optical absorption coefficient that requires thick absorber layers, have prompted the development of other photovoltaic technologies. Compared to first-generation c-Si solar cells, second-generation solar cells typically adopt the inorganic thin-film approach, primarily represented by Copper Indium Gallium Selenide (CIGS) and Cadmium Telluride (CdTe). These technologies have already achieved commercialization, reaching maximum efficiencies of 23.6% and 23.1%, respectively [7,8]. However, Cd is toxic, and In, Ga, and Te are rare metals, which limits their further development [9,10]. The development of environmentally friendly, earth-abundant thin-film materials has rapidly emerged as one of the most prominent research hotspots in contemporary materials science [11,12].

**Figure 1:**
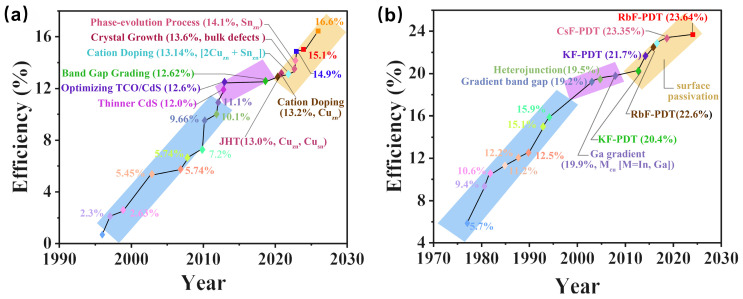
The development of the highest efficiency of (**a**) CZTSSe and (**b**) CIGS thin-film solar cells.

**Figure 2:**
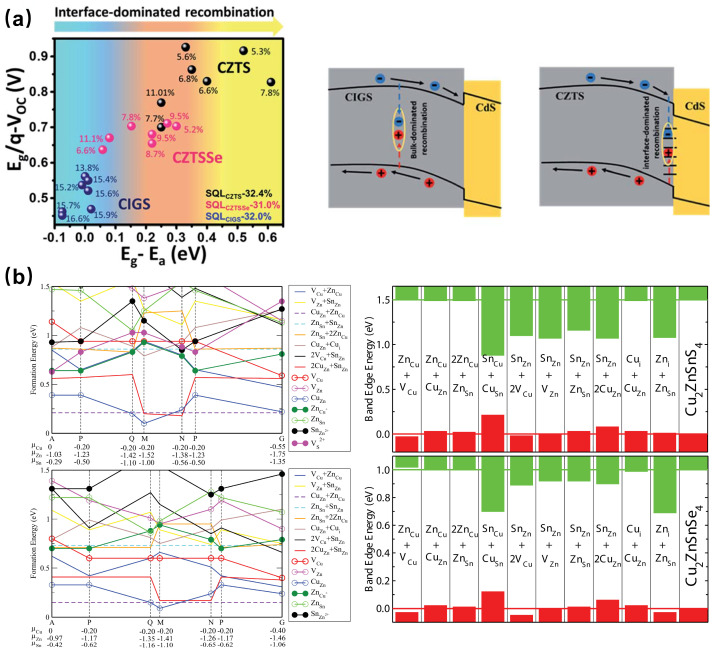
(**a**) Relationship between (Eg-Ea) and Eg/q-Voc for different thin-film solar cells, showing the voltage-loss characteristics of CZTS, CZTSSe, and CIGS solar cells, together with schematic illustrations of bulk-dominated recombination in CIGS and interface-dominated recombination in CZTS. Adapted with permission from [17]. Copyright © 2013, Royal Society of Chemistry. (**b**) Formation energies of low-energy defects and calculated valence and conduction band shifts induced by different defect clusters in Cu_2_ZnSnS_4_ (**top**) and Cu_2_ZnSnSe_4_ (**bottom**). Adapted with permission from [18]. Copyright © 2013, John Wiley and Sons.

Benefiting from its earth-abundant constituent elements, low toxicity, environmental friendliness, low cost, and excellent low-light response, CZTSSe shows strong cost competitiveness and potential for sustainable commercial applications [13]. As a direct bandgap semiconductor, the bandgap of CZTSSe is continuously adjustable in the range of 1.0–1.5 eV by varying the S/Se ratio [14]. The material also has a large absorption coefficient (>10^4^ cm^−1^) [15], and only a thickness of 1 μm is required to achieve sufficient absorption of sunlight. Furthermore, CZTSSe has a crystal structure and optical properties similar to those of commercially proven CIGS. Thus, its research and development can draw on the extensive technical experience accumulated for CIGS devices. Recently, in 2026 the PCE of CZTSSe thin-film solar cells has increased from an initial 0.66% to 16.6% [7]. However, its performance still lags behind that of homologous CIGS devices (Figure 1a) [16]. The disparity between them is usually attributed to the severe carrier recombination within the CZTSSe devices, which results in a large open-circuit voltage deficit (E_g_/q-V_oc_), as well as serious problems of poor back interface and front interface contact. By comparing the performance of commercialized CIGS and CZTSSe solar cells in terms of V_oc_ loss with their bandgaps and recombination activation energies, the intrinsic differences can be clearly revealed [17]. Figure 2a compares the dominant carrier recombination pathways in CIGS, CZTSSe and CZTS technologies. In the case of CIGS, experimental data show that its main recombination path exists in the absorber layer. In contrast, CZTSSe devices mainly suffer from interface-dominated recombination, which primarily takes place at the CZTS/CdS heterojunction due to the “cliff-like” band alignment or interfacial defects, as shown in Figure 2a.

Theoretical calculations and experimental studies can summarize the factors hindering the efficiency improvement of CZTSSe devices into three primary aspects: (i) Crystal quality and bulk defects in the absorber layer: Being a quaternary compound, CZTSSe is made of several constituent elements, which increase the density of intrinsic defects relative to binary or ternary compounds. First-principles computations have shown that the CZTSSe lattice contains various defects, such as antisite defects (Cu_Zn_, Zn_Cu_, Zn_Sn_, Sn_Cu_ and Sn_Zn_), vacancies (V_Cu_, V_Zn_, V_Sn_ and V_(S,Se)_) and defect clusters (V_Cu_ + Zn_Cu_, V_Zn_ + Sn_Zn_, 2Cu_Zn_ + Sn_Zn_ and Zn_Sn_ + 2Zn_Cu_) as shown in Figure 2b [18,19].

Depending on their energy levels within the bandgap, these defects can be classified as shallow or deep-level defects. Shallow defects contribute to carrier concentration and improve the V_oc_, but deep-level defects are non-radiative recombination centers, which seriously affect the performance of devices. Hence, the crucial approach to the optimization of CZTSSe solar cells is suppressing the formation of deep-level defects and rationally controlling shallow defects [20,21]. (ii) Back interface issues: In the typical CZTSSe device structure, Mo is universally employed as the back contact substrate. Presently, many researchers are working on optimizing various deposition techniques and annealing conditions in an effort to develop an ideal CZTSSe absorber layer with no voids and no defects on this Mo substrate [22,23]. Nonetheless, under the high-temperature annealing condition, CZTSSe can react with Mo resulting in the formation of compounds such as Cu_2_(S,Se), Zn(S,Se), Sn(S,Se), Mo(S,Se)_2_. The effect of this reaction is the occurrence of secondary phases, defects and voids at the back interface, thus forming non-ideal ohmic contacts [24,25]. Extensive interfacial reactions result in absorber decomposition and trigger massive non-radiative recombination, a strong negative effect on the device operation [23]. (iii) Front interface issues: The performance of the CZTSSe/CdS heterojunction is primarily limited by two key issues. First, unfavorable band alignment is the direct cause of severe interfacial recombination, and this non-radiative recombination pathway constitutes the primary obstacle limiting the enhancement of the device V_oc_. Second, there is a lack of control in the interdiffusion of the elements at the interface, which further adds to the complexity. Such diffusion is capable of bringing new defect phases and changing the chemical composition of the interface, and has a drastic negative effect on the device’s predictability and stability in the long term.

To address the above issues, researchers have explored various strategies, such as solvent selection, metal precursor modulation, regulation of absorber layer crystallinity, defect state control, and interface engineering [26]. Among these, interface engineering has become an effective method in inhibiting interfacial recombination and enhancing the V_oc_. It has a critical impact on interface-related properties, recombination at various interfaces and carrier collection. To advance the field of interface engineering, this paper first delineates the fundamental device structure and technological development of CZTSSe thin-film solar cells, followed by a systematic summary of the research progress in interface engineering and modulation strategies. Finally, based on the current state of research, this paper explores future directions for interface engineering in CZTSSe thin-film solar cells and proposes strategies for further optimization.

## 2. Device Structure and Development Progress of Kesterite Photovoltaics

### 2.1. Device Structure

**Figure 3:**
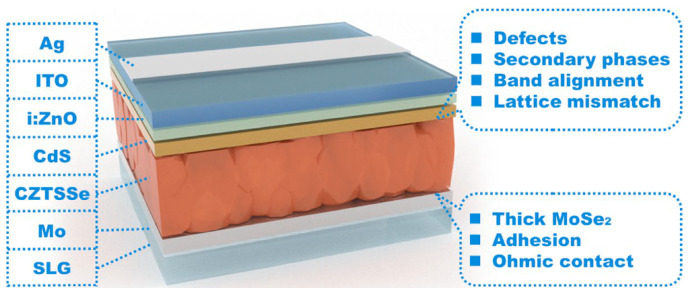
The PV device structure of CZTSSe and the interface problems. Adapted with permission from [27]. Copyright © 2023, Elsevier.

CZTSSe thin-film technology usually has a multi-layered substrate design, which follows the traditional architecture of CIGS cells. That device structure is depicted in Figure 3 [27]. Specifically, its composition layers include: a soda-lime glass (SLG) substrate, a Mo back contact, a p-type CZTSSe absorber layer, an n-type CdS buffer layer, an intrinsic zinc oxide (i-ZnO) layer, an indium tin oxide (ITO) window layer, and a Ag top grid electrode. There are two key interfaces between the front and back contacts in a traditional CZTSSe solar cell: (1) Mo/CZTSSe interface (back interface), which is closely related to the collection efficiency of the diffusion current; and (2) CZTSSe/CdS interface (front interface), which is the key factor determining the charge separation and the final collection efficiency [17].

### 2.2. Development Progress of CZTSSe Thin-Film Solar Cells

The technological advancement of CZTSSe thin-film solar cells can be categorized into three phases:

The first stage before 2000 was the initial exploratory stage of CZTS thin-film studies. This period focused on exploring the properties of CZTS materials and the development of preparation technologies, and laid the foundation for subsequent device applications. In 1988, Ito et al. were the first to prepare the CZTS film using the atomic beam sputtering method, and confirmed that this film possessed p-type semiconductor properties, a high absorption coefficient (1.2 × 10^4^ cm^−1^), and an appropriate bandgap value (1.45 eV), demonstrating the potential for photovoltaic applications. Moreover, the heterojunction prepared with CZTS achieved a V_oc_ of 165 mV [28]. In 1997, a research team led by Katagiri fabricated the first CIGS-like multi-layered structure CZTS solar cell (SLG/Mo/CZTS/CdS/ZnO/AZO/Al) through the vacuum method, and the certified efficiency was 0.66% [29]. This result confirmed the viability of the CZTS system used in photovoltaics and its device architecture is still popular today. Later Friedlmeier et al. enhanced the device conversion efficiency to 2.3% by high-vacuum co-evaporation [30].

**Figure 4:**
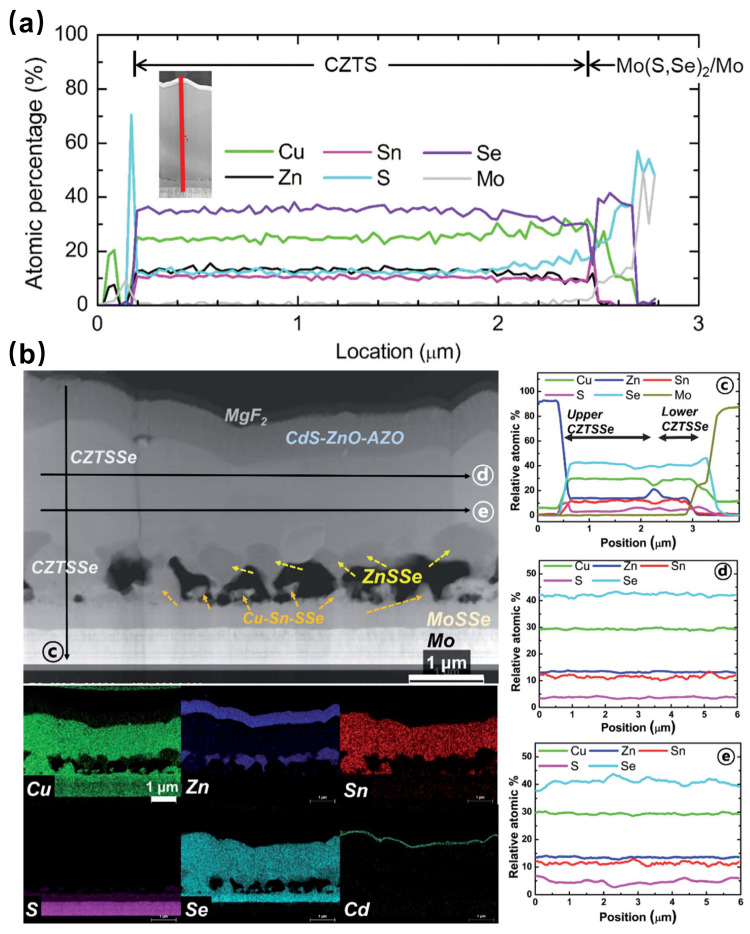
(**a**) EDX analysis across the thickness of the CZTSSe layer in a 12% CZTSSe device (a sister sample to the 12.6% efficiency device). The inset shows the physical path for the EDX scanning in the STEM image. Adapted with permission from [35]. Copyright © 2013, John Wiley and Sons. (**b**) Cross-sectional STEM elemental mapping and EDX line-scan profiles of the champion CZTSSe thin-film solar cell with a certified efficiency of 12.7%. The symbols ⓒ, ⓓ, and ⓔ indicate the corresponding scanning directions in the STEM image [39].

The second stage was from 2000 to 2019. In 2007, Katagiri et al. increased the photoelectric conversion efficiency of CZTS solar cells to 5.74% under Cu-poor Zn-rich conditions by optimizing the proportion of metal cations in the absorber layer [31]. The publication of this key finding laid a foundation for the rapid development of subsequent CZTSSe solar cells, and since then, the efficiency of CZTS solar cells has been continuously improved. In 2009, Guo et al. first prepared CZTS nanoparticles using a non-vacuum hot injection method, then made the nanoparticles into ink and directly coated them on the substrate. After selenization, they successfully fabricated a CZTSSe solar cell with a conversion efficiency of 0.74% [32]. One year later, they successfully increased the cell efficiency to 7.2% [33]. This low-cost, easy non-vacuum method, which was best applied in large-area substrate deposition and also flexible substrate deposition, quickly gained enormous interest in the research community. In 2010, the IBM group reported an efficiency of 9.6% for CZTSSe solar cells made with the hydrazine solution technique. This efficiency was officially recorded in the device efficiency development curve of the National Renewable Energy Laboratory (NREL) [34]. This milestone marked that such cells had officially joined the mainstream list of thin-film photovoltaic candidates. To push the efficiency limits further, the team significantly improved the coating uniformity and bulk film quality by optimizing the hydrazine pure-solution approach. As demonstrated by the energy-dispersive X-ray spectroscopy (EDX) depth profile in Figure 4a, this approach achieved a highly uniform elemental distribution of Cu, Zn, Sn, S, and Se throughout the entire bulk of the CZTSSe absorber layer. Eventually, in 2014, they increased the solar cell’s PCE to 12.6% [35].

Even though the efficiency enhancement experienced a bottleneck period thereafter, the global research teams still achieved a series of innovative results. Wu et al. designed a bandgap grading in the shape of a V using Ag gradient doping and attained an efficiency of 11.2% in CAZTSSe cells [36]. Edgardo et al. introduced high-resistance ZnO layer at the back interface that was effective in suppressing the decomposition reaction between CZTSe and Mo during high-temperature annealing; this enhanced the back interface quality and series resistance, thus improving the performance of the device [37]. In 2018, the team led by Yao optimized the Zn/Sn ratio in the dimethylformamide (DMF) solvent system, and successfully fabricated a CZTSSe solar cell with an efficiency of 8.01%, breaking the previous record for the highest efficiency in the DMF solvent system [38]. In 2019, the DGIST team optimized the annealing process by employing H_2_S gas for sulfo-selenization. As demonstrated by the cross-sectional scanning transmission electron microscopy (STEM) and EDX profiles in Figure 4b, the film exhibits a wide-bandgap ZnSSe secondary phase with a distinct S/(S + Se) depth gradient. Through this approach, back-contact recombination was suppressed by rear passivation and an internal drift field was formed via a back-surface graded bandgap to facilitate carrier collection, leading to a certified efficiency of 12.7% [39].

**Figure 5:**
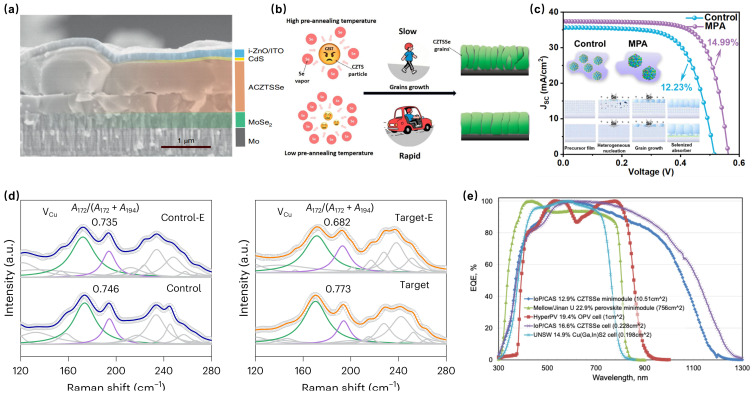
(**a**) Cross-sectional scanning electron microscopy (SEM) image of CZTSSe device of a DMSO-based solution process. Adapted with permission from [40]. Copyright © 2022, Springer Nature. (**b**) A schematic diagram illustrating the influence of pre-annealing temperature on the crystal growth kinetics of the CZTSSe absorber layer. Adapted with permission from [41]. Copyright © 2025, American Chemical Society. (**c**) J-V characteristics and growth mechanism of CZTSSe solar cells: the MPA-treated device has a high efficiency of 14.99% compared to the control. Adapted with permission from [42]. Copyright © 2025, American Chemical Society. (**d**) Raman spectra of the control and Mg-doped CZTSSe films. Adapted with permission from [43]. Copyright © 2025, Springer Nature. (**e**) EQE spectra of the new thin-film cells, including the target CZTSSe cell with a PCE of 16.6%. Adapted with permission from [7]. Copyright © 2026, John Wiley and Sons.

The third stage began in 2020 and continues to the present. In 2022, Xin et al. used a dimethyl sulfoxide (DMSO) solution system and replaced the Sn^2+^ precursor solution with the Sn^4+^ precursor solution to prepare a pure-phase CZTSSe absorber layer with a denser surface and without secondary phases, as shown in Figure 5a. Finally, by combining with heterojunction annealing treatment, the V_oc_ deficit could be reduced to less than 0.34 V resulting in a CZTSSe solar cell with a certified efficiency of 13.0%, which once again broke the world record for the efficiency of CZTSSe solar cells [40]. During 2025, Zhang et al. achieved rapid crystal growth by controlling the pre-annealing temperature. As a crucial thermal treatment prior to sulfurization/selenization, pre-annealing drives elemental diffusion and modulates the phase distribution within the precursor. In this study, the researchers found that a lower pre-annealing temperature improved the microstructure of the precursor, which not only facilitated the penetration of Se but also promoted crystal growth for the Ag-alloyed CZTSSe system during the selenization process (Figure 5b). Therefore, the carrier separation and transport efficiency at the grain boundaries is significantly enhanced, the bulk defects and interfacial defects are reduced, and the carrier recombination in the device is effectively suppressed. Eventually, a flexible CZTSSe solar cell with a certified efficiency of 13.22% was obtained. This work revealed the influence of the pre-annealing treatment process on crystal growth and proposed an effective strategy to improve the quality of kesterite films [41]. Meanwhile, Wu et al. introduced bidentate chelation structured mercaptopropionic acid (MPA) into the air-prepared 2-methoxyethanol (MOE) precursor solution to control the phase transformation from CZTS to CZTSSe in the initial stage of selenization [42]. The strong coordination of the mercapto group and the intermolecular hydrogen bonds helped to form large CZTS colloidal particles and dense precursor films. The reduced number of nucleation sites weakened the interactions with selenium, prolonged the phase evolution process, and promoted the further growth of the bottom small particle layer; therefore, a PCE of 14.99% was achieved (Figure 5c) [42]. The problem of Cu-Zn cation disorder in CZTSSe was addressed by Meng et al., who proposed a strategy of Mg pre-doping to introduce vacancy defects on the surface of the absorber layer. As shown by the Raman spectra and peak intensity fittings in Figure 5d, the Mg-doped sample exhibits a sharp drop in the Raman peak intensity ratio R(A_172_/(A_172_ + A_194_)) from 0.773 to 0.682, whereas the undoped control film barely changes. This contrast directly proves that the combination of Mg pre-doping and etching successfully generates abundant surface V_Cu_. Consequently, this vacancy-assisted strategy significantly reduces the atom migration barrier and enhances the kinetics of Cu-Zn ordering, leading to a certified PCE of 14.9% for a 0.27 cm^2^ device [43]. Currently, this team has further increased the certified efficiency to 16.6%. As shown in the external quantum efficiency (EQE) comparison of various high-performance solar cells in Figure 5e, the CZTSSe device exhibits a high quantum efficiency over a broad spectral range and maintains significant photoresponse in the near-infrared region. Such spectral characteristics highlight its competitive photon-harvesting capability and indicate considerable potential for further performance improvement [7].

## 3. Interface Engineering

### 3.1. Back Interface

#### 3.1.1. Key Issues and Mechanisms at the Back Interface

Although the CZTSSe solar cells directly adopt the mature device structure and Mo back electrode of CIGS, studies have shown that there is an undesirable contact between the Mo electrode and the CZTSSe absorber layer [44]. This undesirable non-ideal ohmic contact between the Mo back electrode and CZTSSe absorber layer is one of the key factors limiting the performance of CZTSSe devices. The main problems are reflected in the following three aspects: the formation of a Mo(S,Se)_2_ intermediate layer, the segregation of secondary phases in the absorber layer, and the creation of interfacial voids.

(1) Formation Mechanisms and Influencing Factors of the Mo(S,Se)_2_ Intermediate Layer

The back interface of Mo/CZTSSe exhibits severe thermodynamic instability during high-temperature selenization. The Mo electrode reacts directly with Se vapor (Equation (1)), and simultaneously, the CZTSSe absorber layer itself decomposes and reacts with Mo (Equation (2)), resulting in the formation of the Mo(S,Se)_2_ interfacial layer [45,46].

(1) $$\mathrm{Mo}+{S(e)}_{2}\left(g\right)\to \mathrm{Mo}{S(e)}_{2}$$

(2) $$2Cu_{2}\mathrm{ZnSn}{S(e)}_{4}+\mathrm{Mo}\to 2Cu_{2}S(e)+2\mathrm{Zn}S(e)+2\mathrm{Sn}S(e)+\mathrm{Mo}{S(e)}_{2}$$

**Figure 6:**
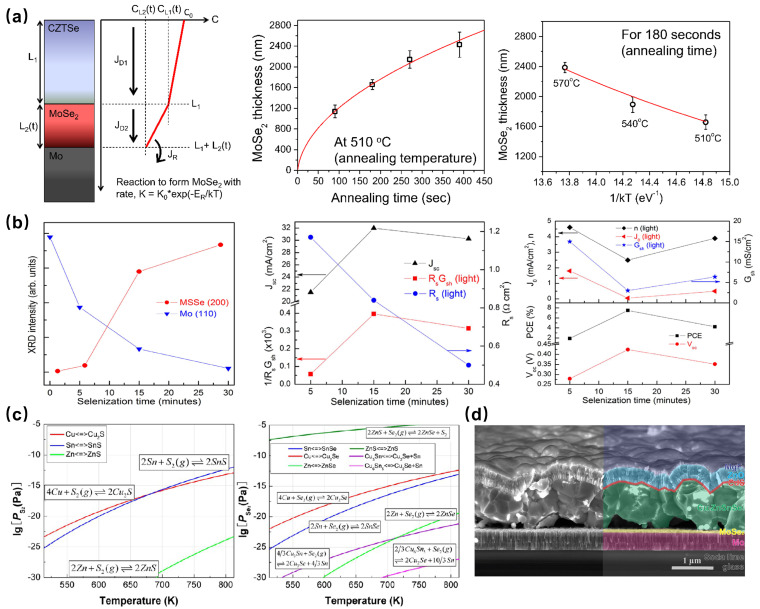
(**a**) Schematic diagram of Se diffusion through CZTSSe/Mo(S,Se)_2_/Mo during high-temperature annealing and the relationship between Mo(S,Se)_2_ layer thickness with selenization time and temperature. Adapted with permission from [47]. Copyright © 2013, AIP Publishing. (**b**) Evolution of X-ray diffraction (XRD) peak intensities of Mo (110) and MSSe (200) reflections, together with key photovoltaic parameters (J_sc_, R_s_, R_sh_, G_sh_, V_oc_, J_o_, n, and PCE), as a function of selenization time. Adapted with permission from [22]. Copyright © 2016, American Chemical Society. (**c**) Temperature dependence of the equilibrium S and Se vapor pressures during the sulfurization and selenization processes, respectively. Adapted with permission from [52]. Copyright © 2014, American Chemical Society. (**d**) Cross-sectional SEM image of CZTSe device with voids. Adapted with permission from [55]. Copyright © 2013, AIP Publishing.

The formation of Mo(S,Se)_2_ is governed by a synergy of factors. In 2013, Shin et al. revealed that its growth kinetics were primarily controlled by the diffusion rate of Se vapor through the CZTSSe film to the Mo surface [47]. In this regard, Shin et al. systematically investigated the growth kinetics of the interfacial Mo(S,Se)_2_ layer in CZTSSe devices. Their results revealed that the thickness of the Mo(S,Se)_2_ layer is positively correlated with the selenization temperature and processing time, as shown in Figure 6a. In 2017, Gao et al. further explored the influence of the surface morphology and structure of the Mo substrate on the preferred orientation and thickness of the Mo(S,Se)_2_ layer. Their results indicated that as the surface roughness of the Mo thin film decreased from Mo-1 (a single-layer structure Mo film prepared under high power of 2.5 W/cm^2^ and low pressure of 0.2 Pa) to Mo-3 (with the same bottom-layer preparation process as Mo-1 and a double-layer structure Mo film deposited under a high pressure of 1.5 Pa and a low power of 0.5 W/cm^2^), the preferred orientation of the Mo(S,Se)_2_ layer shifted from the (100) crystal plane to the (103) crystal plane. Meanwhile, due to the (103) crystal plane orientation of Mo(S,Se)_2_, a tilted Se-Mo-Se sheet-like structure was formed, effectively suppressing the deep diffusion of Se vapor and leading to a significant reduction in layer thickness from 1500 nm to 200 nm. This structural optimization reduced the series resistance (R_s_) of the CZTSSe solar cells from 2.94 Ω·cm^2^ to 0.49 Ω·cm^2^, while the PCE improved from 6.98% to 9.04% [48]. The thickness of the Mo(S,Se)_2_ layer at the back interface has a dual effect on the performance of CZTSSe devices. An appropriate thickness of the Mo(S,Se)_2_ layer is considered beneficial [49]. Cozza et al. utilized SCAPS-1D software simulations to suggest that a 100 nm MoSe_2_ layer can significantly optimize device performance. This is attributed to the fact that appropriate thickness of the Mo(S,Se)_2_ layer can improve the lattice matching and adhesion between CZTSSe and Mo, while optimizing band alignment to facilitate a quasi-ohmic contact, thereby reducing interfacial recombination and enhancing the V_oc_ [50]. However, the instability of the back interface during selenization process and the excessive use of selenium often results in an overgrown Mo(S,Se)_2_ layer. This can lead to an increase in R_s_ and a decrease in the fill factor (FF) [51]. Xiao et al. investigated the effect of selenization time at a selenization temperature of 500 °C on the performance parameters of CZTSSe devices as shown in Figure 6b. The yield of Mo(S,Se)_2_ (MSSe) increased as the selenization time was extended from 5 min to 30 min. Simultaneously, the R_s_ of the devices decreased from 1.17 Ω·cm^2^ to 0.52 Ω·cm^2^, while the composite parameter R_s_G_sh_ (derived from series resistance and shunt conductance) and the short-circuit current density (J_sc_) both exhibited an initial rapid increase followed by a gradual decline. The reason for this phenomenon is that when Mo(S,Se)_2_ is thin, a high-quality ohmic contact can be formed at the Mo/CZTSSe interface, while the improved crystallinity and grain size of the absorber layer with increasing selenization time reduce the resistivity of the CZTSSe thin film. Nevertheless, beyond 15 min of selenization, J_sc_ begins to decrease even as R_s_ continues to drop. The reason for this is that the thicker Mo(S,Se)_2_ layer prevents the holes from transferring from the CZTSSe to the Mo electrode through the quantum tunneling effect, thereby reducing the hole current. Furthermore, a thicker Mo(S,Se)_2_ layer can form a p-n junction with the p-type CZTSSe, creating an energy barrier that hinders the transport of holes and also increases the recombination rate of electron-hole pairs at the Mo/CZTSSe interface. Ultimately, for selenization times exceeding 15 min, increases in shunt conductance (G_sh_), reverse saturation current density (J_o_), and diode ideality factor (n) were observed, leading to reductions in PCE, V_oc_, and J_sc_ [22]. In conclusion, the formation of Mo(S,Se)_2_ is primarily regulated by three key factors: (1) the control of temperature and duration during selenization; (2) the amount of Se; and (3) the surface morphology of the Mo substrate.

(2) Impact of Secondary Phases at the Back Interface

Defects at the back interface originate from complex mechanisms that are not limited to the formation of Mo(S,Se)_2_ but are also intimately linked to secondary phases within the absorber layer and the consequent formation of voids. Yin et al. elucidated the evolutionary pathways of secondary phases during the S/Se process based on thermodynamic studies (Figure 6c) [52]. Their findings indicated distinct disparities in the reactive activities and equilibrium vapor pressures among different elements with S/Se. During the sulfurization process, the equilibrium vapor pressure of ZnS is much lower than that of Cu_2_S and SnS, indicating that the Zn element is more prone to S-oxidation compared to Sn and Cu. Conversely, during the selenization process, although the equilibrium Se vapor pressure curve for Zn selenization lies at a lower level, the conversion rate of ZnS to ZnSe is generally low due to the high energy barrier for S-Se substitution within the pre-formed ZnS lattice. In contrast, the selenization reactions of Cu_2_S and SnS proceed more readily, and they can rapidly produce Cu_2_Se and SnSe. Ultimately, during the complex heating and atmosphere change processes, secondary phases such as ZnS(e) and SnS(e) were widely present in the absorber layer. Notably, the influence of these secondary phases on device performance is dictated not only by the types of their secondary phases, but also by their locations. For instance, Zn(S,Se) located at the back interface increases the R_s_ and impedes carrier transport [53], whereas Zn(S,Se) at the front interface is beneficial for enhancing the PCE [39]. The SnS at the back interface provides a passivation effect, which can facilitate carrier transport, while SnS at the absorber surface is detrimental to the separation and transport of photogenerated carriers [54]. Therefore, accurately identifying the types of secondary phases and their locations is of great significance for comprehensively understanding the mechanism by which they affect device performance. For the back interface, the key strategy should focus on effectively suppressing the formation of detrimental secondary phases such as Zn(S,Se).

(3) Formation of Interfacial Voids

In the current studies, the void structure as shown in Figure 6d was commonly observed between the CZTSSe absorber layer and the Mo layer [55]. The voids located at these interface areas are regarded as one of the main reasons for the low efficiency of the CZTSSe device. Their formation can be mainly explained by the three mechanisms: firstly, during the S/Se thermal treatment process, the thermal diffusion rate difference in the metal elements (particularly Sn, Zn, and Cu) in the CZTSSe film will intensify. This difference leads to the uneven distribution of elements within the absorber layer and triggers the formation and volatilization of the secondary phases SnS(e) and ZnS(e), thereby creating voids. During the thermal treatment process, these voids rupture and eventually form larger voids at the Mo/CZTSSe interface [56,57,58]. Secondly, the selenization/sulfurization temperature is closely related to the formation of voids. Different selenization temperatures significantly affect the crystal structure, elemental composition, morphology of the CZTSSe film, as well as the characteristics of the CZTSSe/Mo interface [59,60]. An excessively high selenization temperature may accelerate the loss of Sn and the formation of volatile secondary phases, thereby increasing the pore density [61,62]. Another important reason for the formation of the voids is the loss of Sn. Compared to other metal elements, the melting point of Sn is lower, and it is prone to partial loss during the thermal treatment process, which directly leads to the formation of voids [63], especially in the Sn-poor areas, where this loss phenomenon is more significant and voids will gradually form at the Mo/CZTSSe interface [64]. The loss of Sn not only leads to the formation of voids, but also aggravates the uneven distribution of elements, further promoting the formation of defects, and ultimately affecting the performance of the device. It was demonstrated by Altamura et al. that the high-density of voids at the interface of Mo/CZTSSe is a structural defect. These voids create additional shunting paths within the device, resulting in a significant reduction in the shunt resistance (R_sh_) and FF of the device, and ultimately leading to a severe decline in V_oc_ and PCE [65]. Thus, the reduction or eradication of these voids can be of significance in improving the performance of these devices.

In summary, the uncontrolled growth of the Mo(S,Se)_2_ layer, the formation of secondary phases, and the presence of interface voids are the three core issues faced by the CZTSSe back interface, which collectively lead to a significant degradation in device performance. To resolve these problems, the recent research has focused on the optimization of interfacial chemical and electrical characteristics by introducing intermediate layers.

#### 3.1.2. Optimizing Back Interface Performance via Intermediate Layer Introduction

In recent years, many researchers have inserted intermediate layers of appropriate thickness between the Mo and the CZTSSe absorber layer to suppress the chemical reactions shown in Equations (1) and (2) at the back interface. This approach suppresses the formation of MoSe_2_ and interfacial voids at the back contact, thereby improving the PCE of the solar cell.

(1) Metal Nitrides

**Figure 7:**
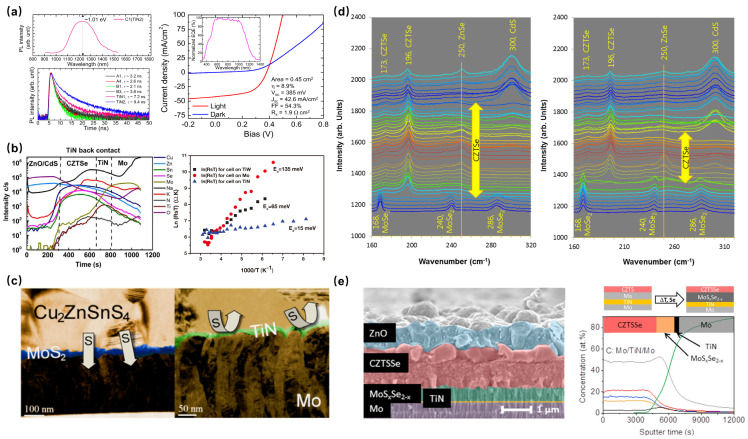
(**a**) PL, TR-PL spectra (left) and J-V characteristics (right) of the CZTSe solar cell with a TiN diffusion barrier. Adapted with permission from [67]. Copyright © 2012, AIP Publishing. (**b**) SIMS of elements Cu, Zn, Sn, Na, K, O and Mo for CZTSe solar cell on TiN back contact and extraction of activation energy from the series resistance for cells on Mo, TiW and TiN. Adapted with permission from [68]. Copyright © 2015, IOP Publishing. (**c**) Schematic illustration of the blocking mechanism of the TiN barrier layer during the high-temperature annealing process [46]. (**d**) Depth profiles of the Raman shift for the CZTSe absorbers formed on bare Mo and MoN barrier substrates. Adapted with permission from [69]. Copyright © 2018, Elsevier. (**e**) Cross-sectional SEM micrograph and SNMS depth profiles of the optimized Mo/TiN/Mo back contact. Adapted with permission from [70]. Copyright © 2016, Elsevier.

In order to resolve the critical issue of interfacial instability between the CZTSSe absorber and the Mo back contact, researchers have conducted intensive research on the introduction of metal nitrides as barrier layers. Among the numerous studies on metal nitrides, the TiN barrier layer has attracted significant attention due to its excellent electrical and optical properties. The work function of TiN is nearly identical to that of Mo, which enables the formation of a good ohmic contact between the absorber layer and the Mo layer [66]. Moreover, the high reflectivity of the TiN layer in the long-wave region contributes to the generation of additional photogenerated carriers within the absorber layer. Early research approaches mainly focused on the chemical inertness of metal nitrides, with the aim of preventing interfacial reactions through physical isolation. Shin et al. systematically demonstrated the effectiveness of TiN [67]. They discovered that after high-temperature annealing, CZTSe would form a MoSe_2_ layer as thick as 1300 nm, which seriously affected the performance. Nevertheless, by inserting a TiN layer just 20 nm thick between the Mo and CZTSe, they successfully limited the MoSe_2_ layer thickness to approximately 220 nm. This study further confirms that the TiN layer effectively acts as a diffusion barrier against Se vapor, thereby suppressing the overgrowth of the MoSe_2_ layer. As shown in the photoluminescence (PL) and time-resolved PL (TR-PL) spectra in Figure 7a, suppressing this interfacial reaction effectively eliminated deep-level defects, thereby generating a sharp near-bandgap emission peak at ~1.01 eV and prolonged the carrier lifetime significantly to 9.4 ns. Consequently, this defect passivation directly resulted in an increase in both J_sc_ and V_oc_, driving the PCE from 2.95% to 8.9%. Oueslati et al. discovered that using TiN as a barrier layer can help decrease the density of voids, inhibit the formation of secondary phases, and enhance the adherence of the back contact. It was calculated that the TiN back contact layer provides the best adhesion with the lowest contact barrier value of about 15 meV compared to 135 meV for Mo, which can be regarded as a good ohmic contact. Furthermore, secondary ion mass spectrometry (SIMS) depth profiles profiling confirms the chemical inertness of the TiN layer against element cross-diffusion, while successfully permitting the beneficial migration of alkali atoms (Na and K) into the absorber (Figure 7b) [68]. Scragg et al. discovered that a TiN barrier layer effectively suppressed the reaction between Mo and CZTS and blocked the reaction between Mo and S during the annealing process (Figure 7c) without affecting the desirable migration of Na out of the substrate. Nevertheless, the contact of TiN/CZTSe has a relatively large R_s_, which implies that additional improvements are needed to develop more suitable barrier materials [46]. Some research groups also used other metal nitrides as intermediate layers in order to improve the interface of Mo/CZTSSe. For instance, Kang et al. used MoN as an intermediate layer to prevent Se vapor diffusion, which could greatly decrease the MoSe_2_ thickness as seen in Figure 7d. However, MoN has a relatively high resistivity, which led to a significant increase in the R_s_ while reducing the PCE [69]. Studies have demonstrated that an ultrathin layer of Mo(S,Se)_2_ is beneficial for the crystallinity and electrical contact of the CZTSSe device. If a barrier layer such as TiN is used to completely prevent Mo(S,Se)_2_ formation, this will actually lead to poor quality of the grain and losses in the V_oc_. Therefore, Schnabel et al. proposed a strategy by depositing an additional Mo layer of variable thickness on top of the TiN layer, which not only inhibits interface decomposition but also improves the stability of device performance. Specifically, an optimal PCE of 7.1% was achieved with a 50 nm thick top Mo layer. The Mo + TiN + thin-Mo structure is to utilize TiN as the primary blocking layer while allowing the sacrificial top Mo layer to react with CZTSSe. As shown by the secondary neutral mass spectrometry (SNMS) depth profiles, this facilitates the formation of a thickness-controllable Mo(S,Se)_2_ interfacial layer, providing a viable back-contact optimization scheme for high-efficiency solution-processed CZTSSe solar cells (Figure 7e) [70].

(2) Metal Oxides

**Figure 8:**
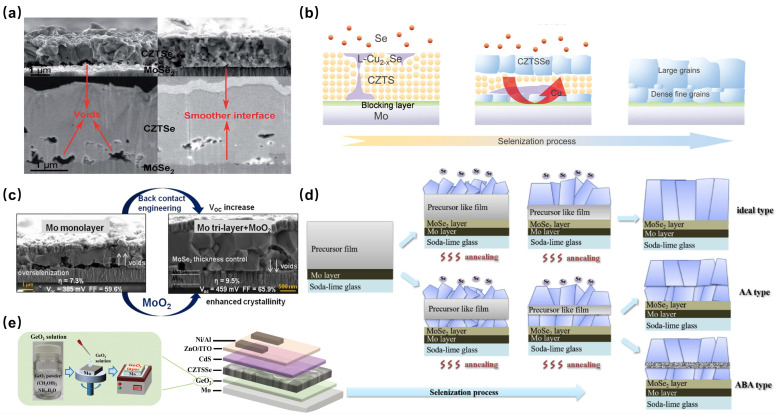
(**a**) Cross-sectional SEM micrographs comparing the morphology of CZTSSe solar cells fabricated with and without a 10 nm ZnO intermediate layer. Adapted with permission from [37]. Copyright © 2013, Royal Society of Chemistry. (**b**) The schematic diagram systematically illustrates the directional migration and diffusion behavior of copper during the crystallization annealing of CZTSSe thin films. Adapted with permission from [74]. Copyright © 2024, John Wiley and Sons. (**c**) The schematic illustrates the regulatory role and underlying mechanism of the MoO_2_ interfacial passivation layer on the photoconversion efficiency of CZTSSe solar cells. Adapted with permission from [75]. Copyright © 2016, Elsevier. (**d**) Schematic illustration of the dynamic evolution mechanism of top-down grain growth in CZTSSe absorber films. Adapted with permission from [77]. Copyright © 2020, Elsevier. (**e**) Schematic diagram of the structure of a CZTSSe device with a GeO_2_ layer prepared by spin-coating a GeO_2_ precursor solution. Adapted with permission from [78]. Copyright © 2022, John Wiley and Sons.

Similarly to the applications in CIGS solar cells, metal oxides such as ZnO, Al_2_O_3_, MoO_x_, and GeO_2_ have demonstrated potential as interface modification layers in CZTSSe photovoltaics. Among them, ZnO is widely used as the intermediate layer material between Mo/CZTSSe due to its high abundance of constituent elements and it is also part of the window layer structure. Marino et al. investigated the role of ZnO as a Mo/CZTSe intermediate layer. Regardless of the introduction of the ZnO intermediate layer, a MoSe_2_ layer with a thickness of up to several hundred nanometers was observed during the annealing process. However, the introduction of a 10 nm ZnO layer effectively inhibits the formation of secondary phases like CuSe, ZnSe and reduces interfacial voids, thereby significantly reducing R_s_ and increasing the PCE from 2.5% to 6.0% (Figure 8a) [37]. Liu et al. achieved control over secondary phases by adjusting the annealing temperature in the presence of a ZnO layer at the back interface, ultimately reducing the size and density of voids at the Mo/CZTSSe interface. As a result, the V_oc_ improved from 324 mV to 641 mV and the J_sc_ improved from 10.8 mA·cm^−2^ to 15.97 mA·cm^−2^ [71]. In addition, Al_2_O_3_ has also been extensively studied due to its excellent chemical stability and insulation properties. In 2017, Liu et al. regulated back-contact self-organized nanopatterns by introducing an Al_2_O_3_ intermediate layer at the Mo/CZTS interface. Before sulfidation, the continuous Al_2_O_3_ intermediate layer can completely isolate CZTS from Mo, inhibiting the harmful interfacial reactions and preventing the formation of secondary phases and thick MoS_2_ layers. During the high-temperature sulfurization process, the Al_2_O_3_ layer converted into discontinuous aggregates, which could form nanopatterned Al_2_O_3_ arrays between CZTS and MoS_2_ with openings for their connection. Meanwhile, the MoS_2_ initially formed in the opening regions acts as a barrier layer, further avoiding direct contact between CZTS and Mo. By utilizing this reaction route to eliminate phase segregation and voids in the back contact area, the team achieved a 9.26% efficiency for a 0.237 cm^2^ cell [72]. Another important role of Al_2_O_3_ was proposed by Kim et al. in 2019 [73] during the synthesis of CZTSSe through a metal precursor approach. In this process a liquid Cu-Sn alloy will be formed, which exhibits wettability on traditional Mo surfaces, leading to residue accumulation in the back contact region and the subsequent formation of harmful Cu-Sn-SSe secondary phases. In contrast, the Al_2_O_3_ surface exhibits extremely poor wettability with the liquid metal, which forces the Cu-Sn alloy to migrate upward during crystallization, thereby avoiding secondary phase formation at the back interface [73]. Li et al. recently used atomic layer deposition (ALD) to fabricate an ultrathin Al_2_O_3_ barrier layer on the Mo to prevent the diffusion of Cu at the back interface, transforming the absorber layer structure from a three-layer structure to a two-layer structure (Figure 8b). The element distribution became more uniform and the defect density of the absorber layer was significantly reduced. Finally, a PCE of 14.9% was achieved with a V_oc_ of 576 mV, and this provides a new direction for performance breakthroughs in multi-element semiconductor photovoltaic devices [74]. Lopez-Marino et al. investigated the influence of MoO_2_ as intermediate layer at the Mo/CZTSe interface on the device performance. They found that MoO_2_ could effectively prevent the formation of MoSe_2_. They prepared a substrate with a structure of Mo/MoO_2_/Mo, successfully controlling the thickness of MoSe_2_ to be below 100 nm, and MoO_2_ could significantly affect the growth orientation of MoSe_2_, ultimately improving the grain size of the absorber layer. The PCE of the device increased from 7.2% to 9.5%, as shown in Figure 8c [75]. In 2019, Wu et al. conducted a study on the distribution of microscopic elements after inserting the MoO_3_ layer. They discovered that the MoO_3_ layer was not destroyed following the high-temperature annealing and the thickness of the MoSe_2_ layer was decreased. When the 10 nm MoO_3_ layer was inserted, the efficiency was increased from 9.02% to the optimal performance of 11.37% [76]. To further elucidate the mechanism by which the MoO_3_ promotes grain growth, Yu et al. introduced an in situ grown MoO_3_ layer and detailed the crystal growth during the early stages of selenization. They discovered that the direct reaction between CZTS and Mo was the root cause of multi-layer crystallization. Introducing the MoO_3_ isolation layer effectively blocked the initial reaction at the back interface, thereby eliminating the crystal nucleation centers on it. This forced the CZTSSe crystals to grow only from the front surface in a self-top-down direction (as shown in Figure 8d), ultimately forming a single crystal grain layer structure throughout the entire absorber layer. Based on this controlled CZTSSe absorber layer, a certified PCE of 11.68% was achieved, offering a key solution for high-efficiency DMSO-processed CZTSSe cells [77]. Among the various metal oxides used for back interface improvement, the application of GeO_2_ demonstrates an efficient strategy. Wang et al. introduced a thin film of GeO_2_ on the Mo substrate, which exhibits bidirectional diffusion property during the high-temperature selenization process, as shown in Figure 8e. Further study found that in the process of selenization, one part of it moved upward to the absorber layer and combined with Se to form Ge-Se liquid flux, thereby promoting nucleation and grain growth, resulting in a CZTSSe film with a smoother surface and fewer voids. In addition, both bulk defects and band tailing have been significantly suppressed, while the hole concentration has increased, thereby reducing the recombination process and achieving a better Fermi-level splitting. The other part moves downward to the MoSe_2_ layer to increase the work function, which increases the band bending at the back interface and enables better separation of photogenerated carriers. The PCE of the champion device reached 13.14%, and the V_oc_ was 547 mV [78].

In conclusion, the use of metal oxides for interface modification is an effective strategy to regulate the back interface properties of CZTSSe devices. These oxide intermediate layers effectively mitigate recombination losses at the back interface by blocking harmful elemental diffusion, suppressing secondary phase formation, and optimizing MoSe_2_ growth. Additionally, by controlling crystallization kinetics and introducing functional elements such as Ge, bidirectional optimization of the bulk and interface was achieved; these strategies significantly improve absorber crystal quality and band alignment. These improvements optimize the V_oc_ and FF, providing robust process support for realizing high-efficiency, low-cost CZTSSe thin-film solar cells.

(3) Metal Sulfides

**Figure 9:**
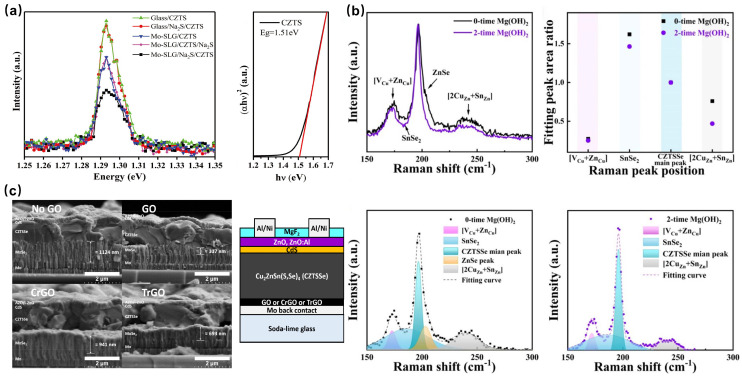
(**a**) PL spectra of CZTS films with different substrate and interface modifications and calculated bandgap of CZTS films on Na_2_S-modified Mo-coated substrates. The red line represents the linear fitting used to determine the optical bandgap. Adapted with permission from [81]. Copyright © 2018, John Wiley and Sons. (**b**) Raman phase analysis and structural evolution of the CZTSSe back interface. Adapted with permission from [82]. Copyright © 2025, IOP Publishing. (**c**) Cross-sectional SEM images and schematic of the CZTSSe solar cells fabricated with different graphene oxide intermediate layers [84].

Metal sulfides are also frequently used as interlayers to optimize the Mo/CZTSSe interface. Chen et al. employed SnS as an interlayer material to improve the quality of the Mo/CZTSSe interface. On the one hand, SnS prevents direct contact between CZTS and Mo, thereby suppressing the excessive formation of MoS_2_ and the decomposition of CZTS; on the other hand, SnS serves as a precursor for the absorber layer, reacting with Cu_2_S and ZnS to form CZTS. The reaction process is shown in Equations (3)–(5) [79],

(3) $$\mathrm{SnS}+S\to (Sn_{2}S_{3})\to \mathrm{Sn}S_{2}$$

(4) $$Cu_{2}S+\mathrm{Sn}S_{2}\to Cu_{2}S{\mathrm{nS}}_{3}/Cu_{4}S{\mathrm{nS}}_{4}/Cu_{2}Sn_{4}S_{9}$$

(5) $$Cu_{2}\mathrm{Sn}S_{3}+\mathrm{ZnS}\to Cu_{2}\mathrm{ZnS}{\mathrm{nS}}_{4}$$

as can be seen from the reaction equations, these reactions reduce the formation of secondary phases (Cu_2_S, ZnS) at the back interface and effectively minimize the formation of voids at the back interface. It has also been verified that SLG as a substrate can significantly enhance the performance of the CZTSSe solar cells [80]. Therefore, Gu et al. proposed a self-depleted back contact modification layer, and found that the introduction of Na_2_S could stabilize the CZTS/Mo interface and protect the Mo contact layer through two competition effects: one is the reaction between Na_2_S and the Mo contact layer, and the other is the competition between CZTS and H_2_S. As illustrated in Figure 9a, the enhanced PL response of the modified films indicates reduced defect-assisted recombination, while the bandgap analysis reveals that the electronic structure of CZTS remains largely unchanged. These results suggest that the performance improvement primarily arises from interfacial defect passivation and enhanced carrier collection, leading to increases in both Voc and Jsc [81]. The most specific feature of the utilization of the metal sulfides as intermediate layers is the possibility to be involved in the crystallization of the absorber layer as a reactant to exclude the introduction of the impurities or disadvantageous aspects related to the foreign material.

While the performance of devices incorporating metal sulfides as the intermediate layer remains modest to date, metal sulfides can act as reactive precursors to participate in the crystallization of the absorber layer. Specifically, they can suppress the formation of Mo(S,Se)_2_ and voids, while also effectively precluding the introduction of other adverse factors at the back interface. This is the greatest advantage of using metal sulfides as the intermediate layer material.

(4) Other Intermediate Materials

**Figure 10:**
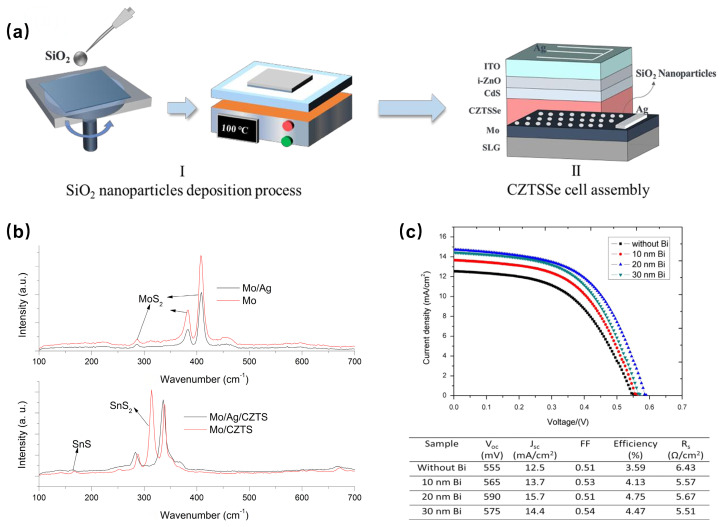
(**a**) Schematic flowchart illustrating the synthesis of SiO_2_ nanoparticles and the device structure of a CZTSSe solar cell featuring a SiO_2_ nanoparticle-based porous insulated contact (PIC) architecture [85]. (**b**) Raman spectroscopic analysis of the CZTS/Mo interface with and without 20 nm Ag intermediate layer. Adapted with permission from [86]. Copyright © 2014, AIP Publishing. (**c**) J-V characteristics of CZTS thin-film solar cells with different thicknesses of pre-deposited Bi, the V_oc_, J_sc_, η, FF and R_s_ are given. Adapted with permission from [87]. Copyright © 2015, Elsevier.

In addition to the materials summarized above, researchers have successfully employed a variety of other materials, including metal hydroxides, carbon-based materials, SiO_2_, etc., to engineer and regulate the Mo/CZTSSe interface. Taking the Mg(OH)_2_ as an example, Zhang et al. developed an ultrathin Mg(OH)_2_ intermediate layer between the Mo substrate and the CZTSSe absorber layer through spin-coating. This Mg(OH)_2_ intermediate layer can serve as an effective physical barrier, which suppresses the uncontrolled growth of MoSe_2_ and decreases the formation of interfacial secondary phases. More significantly, in the high-temperature selenization, Mg^2+^ will diffuse into the CZTSSe lattice and replace the Zn^2+^. This ion exchange is not only an effective way to suppress harmful defect clusters (2Cu_Zn_ + Sn_Zn_) but also promotes the formation of beneficial defect clusters (V_Cu_ + Zn_Cu_). As a result, this reduces the band tailing states related to the presence of defects, as illustrated in Figure 9b [82]. Another popular type of intermediate layer has been carbon-based materials because of their chemical inertness and good electrical conductivity. The study by Zhou et al. proved that a single ultrathin C layer can play a crucial role. Even though it is not able to entirely avoid the formation of MoS_2_, there are voids in interfaces caused by the volatilization of SnS(e) that are adsorbed and filled by the carbon layer, restoring electrical connection between the absorber and the back electrode. This strategy effectively reduces the interfacial contact resistance, greatly decreases the R_s_ and increases the J_sc_ of the CZTSSe device [83]. Based on this, as an advanced carbon material, graphene oxide (GO) and its reduced graphene oxide (RGO) provide a greater degree of control for back interface modification. Jeong et al. have confirmed that by introducing GO or RGO intermediate layers, the formation of MoSe_2_ and the decomposition of CZTSSe can be effectively suppressed. The CZTSSe solar cell structure and the profiles of SEM cross-sectional cell morphologies were shown in Figure 9c. The chemically reduced CrGO, which positively affects the fill factor, resulted in a conversion efficiency of 6.3%, providing a reference to intermediate layer design in CZTSSe solar cells [84]. In 2025, Lin et al. developed a simple control strategy for incorporating SiO_2_ nanoparticles on the Mo substrate using the spin-coating method (Figure 10a). This method achieves the formation of discrete local contact structures by leveraging the insulating properties of SiO_2_, thereby enhancing the crystallinity of the absorber layer, reducing the density of interfacial defects and specifically suppressing the harmful [2Cu_Zn_ + Sn_Zn_] deep-level clusters. This approach at the same time is effective at inhibiting nanoparticle agglomeration, and maintains the optimal hole–transport distances, thereby obtaining a more efficient passivated interfacial contact (PIC) structure, and improve the back-contact quality, increasing the PCE of CZTSSe solar cells from 9.03% to 10.72% [85]. Certain metallic materials have also been successfully employed as intermediate layers. For instance, Ag as an intermediate layer has a similar role to ZnO. It reduces the interfacial voids by preventing the formation of SnS_2_ and, at the same time, it can prevent the generation of sulfur vapor to reduce the formation of MoS_2_. The SnS_2_ characteristic peaks will not exist in the Ag-modified samples as shown in Figure 10b, and the intensity of the MoS_2_ characteristic peaks was significantly reduced. These findings offer strong proof of the fact that a Ag intermediate layer can be used effectively to inhibit the presence of both SnS_2_ and MoS_2_ secondary phases [86]. Recent work by Tong et al. demonstrated that introducing a pre-deposited thin Bi interlayer on Mo-coated glass effectively optimizes both the bulk absorber layer morphology and the back-contact properties. Since metallic Bi melts at a relatively low temperature (270 °C), it can exist in a liquid form during the high-temperature S/Se process and act as fluxing agent. This liquid-phase environment significantly promotes the crystallization process of CZTS with the formation of larger grain size and fewer grain boundaries. This minimizes carrier recombination across grain boundaries in the absorber layer and at the same time reduces the device R_s_. The J-V curves of CZTS solar cells at different pre-deposited Bi layer thicknesses are shown in Figure 10c, and the corresponding device parameters are summarized in the inset table of the figure. Compared to the reference devices, those integrating the pre-deposited Bi layer showed pronounced increases in the V_oc_, J_sc_, and PCE [87].

Although various interface engineering strategies such as metal hydroxides, carbon-based materials, oxides, and metal thin films have achieved positive progress in inhibiting interface side reactions, optimizing energy level matching, and improving crystal quality, the PCE of current modified devices still has a significant gap compared to the theoretical efficiency limit of CZTSSe solar cells. Future research still needs to develop new composite intermediate layer materials with high stability and low resistance loss, in order to further break through the efficiency bottleneck.

### 3.2. Front Interface

#### 3.2.1. Key Issues and Mechanisms at the Front Interface

The p-n heterojunction interface composed of the CZTSSe absorber layer and the n-type CdS buffer layer is the most important pathway in the photogenerated carrier transport and is crucial for the separation and collection of photogenerated charges. Microscopic characteristics of the interface directly determine the final device performance [24,88].

**Figure 11:**
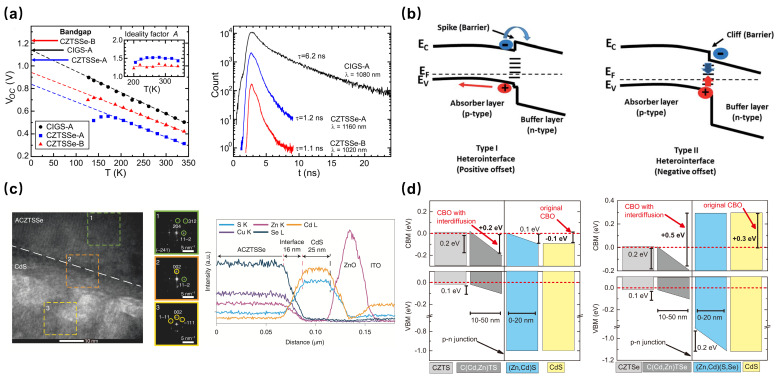
(**a**) Temperature dependence of the V_oc_ for CZTSSe and CIGS solar cells, alongside an analysis of minority carrier lifetimes. Inset: temperature dependence of the ideality factor for the CZTSSe solar cell. Adapted with permission from [89]. Copyright © 2010, AIP Publishing. (**b**) Schematic illustrations of Type I (**left**) and Type II (**right**) heterojunction interface structures (Note: E_C_, E_F_, and E_V_ denote the conduction band minimum, Fermi level and valence band maximum, respectively). Adapted with permission from [17]. Copyright © 2013, Royal Society of Chemistry. (**c**) High-resolution transmission electron microscopy (HRTEM) images and corresponding fast Fourier transform (FFT) patterns of the heterojunction interface, illustrating the Zn/Cd interdiffusion induced by the CBD of CdS. Adapted with permission from [40]. Copyright © 2022, Springer Nature. (**d**) Evolution of band alignment at the CZTS(Se)/CdS interface following significant elemental interdiffusion. Adapted with permission from [99]. Copyright © 2017, Elsevier.

The CZTSSe solar cells have a much higher E_g_/q-V_oc_ than CIGS solar cells even though they follow the mature CIGS device architecture. To compare the main differences in the recombination pathways of these two types of solar cells, Gunawan et al. analyzed the temperature dependence of the V_oc_ and the results are shown in Figure 11a [89]. The analysis showed that the recombination process of CIGS devices mainly occurs inside the bulk material, and recombination at the interface can be disregarded. In contrast, CZTSSe solar cells exhibit severe interfacial recombination. This difference in this interfacial recombination mechanism directly results in significant variations in the minority carrier lifetime of the two devices. Therefore, the severe interfacial recombination at the CZTSSe/CdS heterojunction is the primary cause of V_oc_ deficit. From the above research, the factors influencing the CZTSSe/CdS interface are manifested in the following two aspects: (1) non-ideal band alignment at the heterojunction; and (2) the high density of interfacial defects [90]. Thus, optimizing the band alignment of the heterojunction, passivating interfacial defects and inhibiting the formation of secondary phases can effectively enhance the performance of the device.

(1) Band Alignment of the Heterojunction

The band alignment at the CZTSSe/CdS heterojunction is a core factor determining solar cell performance. The CdS buffer layer has a wide bandgap of about 2.4 eV [91], and the bandgap of the CZTSSe absorber layer can be adjusted within the range of 1.0–1.5 eV. When the bandgap of the absorber layer changes, the energy band alignment formed by its contact with CdS will also change accordingly. Figure 11b shows the two energy band distributions at the interface of the CZTSSe/CdS heterojunction: Type I (“spike-like”) and Type II (“cliff-like”) [17]. When the conduction band minimum (CBM) of the absorber layer is lower than that of the buffer layer, a “spike-like” type band structure will be formed. To achieve efficient charge transport, the ideal conduction band offset (CBO) range should be controlled within 0–0.4 eV [92]. The spike structure in this range causes a small energy barrier at the interface, effectively blocking most charge carriers from flowing towards the interface, thus suppressing interfacial recombination and enhancing V_oc_. Nevertheless, when the CBO is greater than 0.4 eV, the excessively high barrier will prevent the movement of electrons, causing the loss of current [93]. The conduction band position of CZTSSe is relatively high, when combined with CdS to form a p-n junction, it will result in a “cliff-like” structure [17]. The “cliff-like” band alignment is unfavorable to device performance due to the narrowing of interfacial bandgap As a consequence, electrons in CdS and holes in CZTSSe can recombine through interface defect levels, thus reducing the V_oc_, FF, and PCE of the device [94].

Studies show that the “spike-like” structure with a CBO value in the range of 0–0.4 eV is significantly more effective in suppressing interfacial recombination compared to the “cliff-like” structure, making it an ideal arrangement for achieving high-efficiency devices.

(2) Front Interfacial Defects

The multinary composition of CZTSSe materials results in a wide variety of complex lattice defects, which are obvious at the film surfaces and interfacial regions. When photogenerated carriers pass through the p-n junction, these defects will accelerate the recombination of photogenerated carriers, reduce the separation efficiency of photogenerated carriers, and consequently lead to a decrease in the V_oc_. The Cu_Zn_ antisite defect is regarded as a major factor restricting the improvement of the performance of CZTSSe solar cell. This is attributed to the similar ionic radii of the Cu^+^ (0.77 Å) and Zn^2+^ (0.74 Å); the Cu_Zn_ antisite defect has a low formation energy, resulting in a high concentration within the absorber layer [95]. However, there is a large number of Cu_Zn_ antisite defects and V_Cu_ in the crystal phase, which result in the p-type semiconductor characteristics of the CZTSSe films. The high concentration of Cu_Zn_ antisite defects at the CZTSSe/CdS interface causes the Fermi level pinning, resulting in only a small band bending in the absorber layer, which is not conducive to the improvement of V_oc_ [94,96,97]. Furthermore, a single Cu_Zn_ defect is prone to form [2Cu_Zn_ + Sn_Zn_] defect clusters with other defects, such as Sn_Zn_. These defect clusters will introduce deep-level defects in the bandgap, which act as efficient non-radiative recombination centers and induce severe band tailing. In phase-separating solvent systems prone to secondary phase formation, SnSe tends to form on grain surfaces during selenization, causing local Sn enrichment. Although SnSe evaporates during subsequent high-temperature processing, it often leaves behind a high density of Sn-related defects on the surface [98]. Research also shows that the chemical bath deposition (CBD) of CdS can induce interdiffusion of Zn and Cd at the front interface, causing defect formation and lattice mismatch (Figure 11c) [40]. The element diffusion at the interface of the CZTSSe/CdS heterojunction also leads to changes in the band alignment (Figure 11d). The Cd diffuses into the CZTS layer and Zn into the CdS layer, resulting in the formation of a thin C(Cd,Zn)TS layer on the CZTS side, while a (Zn,Cd)S thin layer will form on the side of the CdS buffer layer. In the absence of elemental interdiffusion, the CZTS/CdS interface exhibits a CBO of −0.1 eV, presenting a “cliff-like” band alignment that is unfavorable for device performance. Nevertheless, after the element interdiffusion occurs, the band position is transformed into a desirable profile in the form of a spike with a CBO of +0.2 eV. However, for the CZTSe/CdS heterojunction, the inter-element diffusion will cause the CBO to increase from 0.3 eV to 0.5 eV forming a photogenerated carrier transport barrier, which is harmful to the performance of the devices. These results indicate that the interfacial elemental interdiffusion has an impact on the value of valence band offset (VBO) as well as CBO values [99]. Furthermore, the Se dangling bonds present on the surface of the absorber layer as well as V_Se_ will also form recombination centers, thereby affecting the performance of the device [100].

Based on the above analyses, to further optimize the CZTSSe/CdS interface and break through efficiency bottleneck, current mainstream research focuses on post-treatment technologies, which mainly include alkali metal post-deposition treatment (PDT), heterojunction heat treatment and passivation layers. In the following section, these interface engineering strategies will be reviewed systematically.

#### 3.2.2. Alkali Metal Post-Deposition Treatment

**Figure 12:**
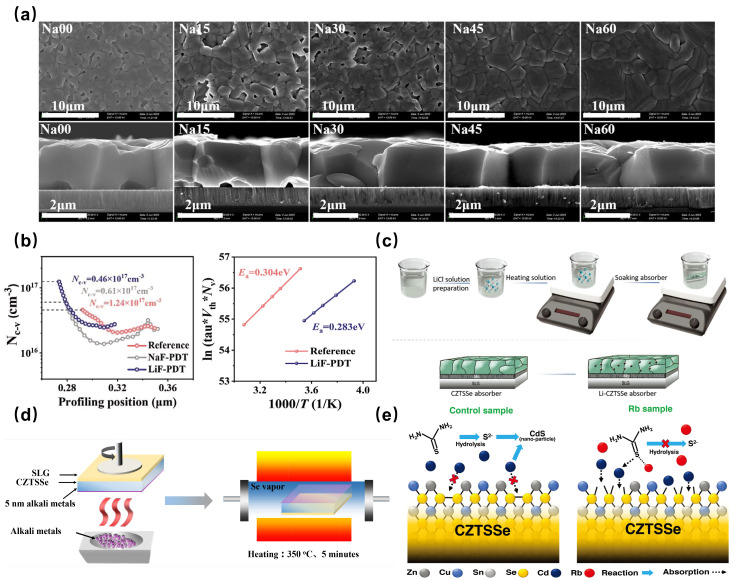
(**a**) SEM images of the annealed absorber layers with varying Na doping concentrations. Adapted with permission from [105]. Copyright © 2023, John Wiley and Sons. (**b**) Calculated free carrier densities and Arrhenius plots derived from the deep-level transient spectroscopy (DLTS) peaks for the reference, NaF-PDT and LiF-PDT devices. Adapted with permission from [108]. Copyright © 2020, Elsevier. (**c**) Schematic illustration of the LiCl solution preparation process and the Li-PDT methodology, alongside a comparison between undoped and Li-doped CZTSSe absorbers. Adapted with permission from [109]. Copyright © 2021, John Wiley and Sons. (**d**) Schematic diagram of the alkali metals post-deposition treatment procedure. Adapted with permission from [110]. Copyright © 2021, Royal Society of Chemistry. (**e**) Schematic diagram depicting the impact of Rb^+^ on the CBD process of the CdS buffer layer. Adapted with permission from [111]. Copyright © 2023, John Wiley and Sons.

PDT is a mature technique originally developed in the CIGS solar cell field and is mainly used to adjust the band structure of the absorber layer/CdS heterojunction interface [101,102,103]. Researchers have applied alkali metal PDT to CZTSSe solar cells with similar efficiency improvements in recent years. Due to their smaller ionic radii and lower diffusion barriers, Na and Li can more effectively diffuse into the CZTSSe lattice and preferentially occupy the Cu cation sites, thereby inhibiting the formation of harmful deep-level defects. In order to study the effect of Na doping on the surface characteristics of the absorber layer, the growth of a buffer layer and device performance, in 2021, Sun et al. prepared Na-doped CZTSSe through a solution approach. Their structural analysis revealed that Na incorporation shifts the XRD peaks position to smaller angles, indicating that the lattice spacing increases because the radius of Na is larger than that of the cations in CZTSSe. Concurrently, Na doping induces a more Cu-poor and Zn-rich environment at the absorber layer surface, and the inherent point defects on the surface are optimized simultaneously: the favorable [V_Cu_ + Zn_Cu_] defect cluster density increases, and the density of the harmful [Cu_Zn_ + Zn_Cu_] and [2Cu_Zn_ + SnZn] defect clusters decreases. In addition, light Na doping in CZTSSe solar cells will increase the quasi-Fermi level difference (E_Fn_-E_Fp_), and V_oc_ will be significantly enhanced [104]. In 2023, Dong et al. achieved in situ Na doping of CZTSSe solar cells by magnetron sputtering of a NaF-containing CZTS target. They further revealed the regulatory mechanism of Na on grain boundaries and band structure. An analysis of the absorber layer surface and cross-sections by SEM after annealing with various doping concentrations (Figure 12a) showed a great enhancement of grain size and a decrease in the voids density with the increase in the concentration of Na. At the same time, Na doping has optimized the CBO at the interface and reduced carrier recombination at the CZTSSe/CdS interface, ultimately achieving an efficiency of 11.18% [105]. In 2024, Siqin et al. suggested a two-passivation strategy, which involved adding Na on the surface of the absorber layer to passivate the CZTSSe/CdS interface, and using ultraviolet (UV) irradiation to treat CdS to passivate the CdS/ZnO interface. These two complementary passivation strategies achieved a synergistic effect, ultimately leading to the preparation of the best device with a PCE of 10.69%, and it also exhibited remarkable long-term stability [106]. Accumulating evidence suggests that Li is the most effective element among the alkali metal group. Compared to Na, Li has a smaller ionic radius and a significantly lower Li_Cu_ substitution energy. On one hand, this low substitution energy can effectively reduce the densities of Cu_Zn_ defects and [2Cu_Zn_ + Sn_Zn_] defect pairs. On the other hand, Li also exhibits superior performance in experiments. Nevertheless, all reported Li^+^-doped CZTSSe solar cells based on in situ doping and pre-deposition doping strategies commonly suffer from massive Li losses and Na rejection due to the adoption of SLG substrates. In 2020, Guo et al. reported a co-selenization approach using LiF and Se sources. By regulating the band alignment of the CZTSSe/CdS interface through this Se and LiF co-selenization process, the device achieved a PCE of 11.6% with a V_oc_ deficit as low as 0.583 V [107]. Zhao et al. achieved synergistic regulation by introducing LiF-PDT on the Ag-substituted CZTSSe absorber layer. Their experiment showed that Li introduction increased the p-type carrier concentration from 4.60 × 10^16^ cm^−3^ to 1.24 × 10^17^ cm^−3^, bulk charge collection was improved, the defect activation energy (E_a_ from 0.304 eV to 0.283 eV) and reversed the electric field polarity at the grain boundaries (GBs), ultimately increasing the PCE from 9.2% to 10.23% (Figure 12b) [108]. Also, Hao et al. presented a non-traditional alkali metal PDT method, in which the selenized films were dipped into hot solution of LiCl, achieving uniform doping of Li within the crystal (Figure 12c). Li doping was very effective in enhancing the carrier density of the film. Meanwhile, the high density of Li_Zn_ prevented activation of Cu_Zn_ defects, significantly improving the p-type doping density and this reduced recombination in space charge region. As a result, the CZTSSe device efficiency increased to 10.7% [109]. Conversely, owing to their much larger ionic radii compared with Cu^+^ in the CZTSSe lattice, heavy alkali metals are hindered from effectively penetrating into the crystal bulk. Therefore, their underlying mechanisms are predominantly manifested in the modification of GBs and surfaces. A dual alkali metals synergistic incorporation project combined light and heavy alkali metals by post-depositing CsF after NaF-PDT procedure to improve the efficiency of CZTSSe solar cells was used by Wu et al., as shown in Figure 12d. They found different functionalities for each element by applying 5 nm of NaF-PDT, then subsequently 5 nm of CsF-PDT. The Na atoms are randomly dispersed both in grain interior (GI) and GBs, significantly increasing the acceptor concentration and passivating the deep-level defects to enhance V_oc_. Meanwhile, the Cs atoms focus on increasing the potential at the GBs, and are more inclined to improve FF. This ultimately achieves a high efficiency of 12.16% [110]. Xu et al. suggested a heterojunction reconstruction strategy assisted by Rb^+^, and revealed the unique dual regulatory mechanism of Rb^+^. On the one hand, the Rb^+^ plays a crucial role in breaking up the Se–Se bond on the surface of the CZTSSe film through Rb–Se interactions. This interaction not only passivates the detrimental zero-valence selenium defect (Se^0^) but also creates a more reactive surface for the epitaxial growth of the CdS layer. On the other hand, Rb^+^ can coordinate with the thiourea (TU) present in the solution, inhibiting the direct hydrolysis of TU and consequently leading to improved ion-by-ion deposition of the CdS layer with more uniform and compact morphology (Figure 12e). These results have a positive impact on the quality of the heterojunction interface and could ultimately achieve a PCE of 12.8% for flexible CZTSSe solar cells [111].

In conclusion, the PDT with alkali metals, leveraging its mature experience in the CIGS system, has evolved into a key process for enhancing the performance of CZTSSe photovoltaic devices.

#### 3.2.3. Heterojunction Heat Treatment

**Figure 13:**
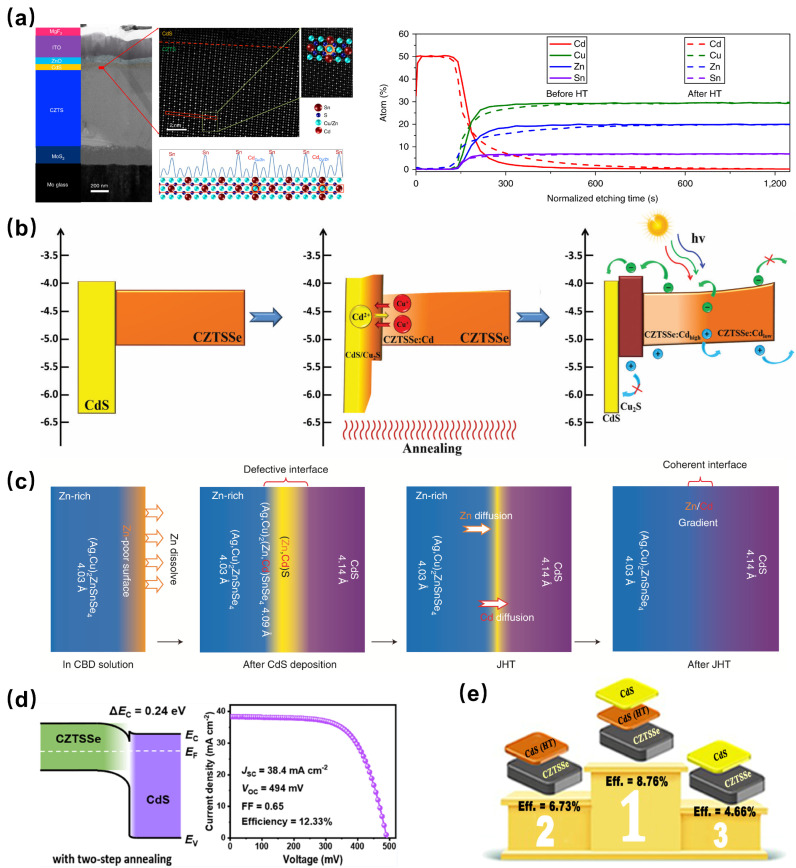
(**a**) Schematic illustration of the band alignment at the CZTSSe/CdS interface with and without heterojunction heat treatment. Adapted with permission from [114]. Copyright © 2018, Springer Nature. (**b**) Band diagram of the CdS/CZTSSe heterojunction (**left**), schematic illustration of Cu^+^ and Cd^2+^ ion diffusion during heterojunction annealing (**middle**), and energy spectrum of CdS/Cu_2_S/CZTSSe:Cd_high_/CZTSSe:Cd_low_ (**right**). Adapted with permission from [116]. Copyright © 2022, Royal Society of Chemistry. (**c**) Illustration of elemental migration at the ACZTSSe surface and ACZTSSe/CdS interface during CBD and JHT processes. Adapted with permission from [40]. Copyright © 2022, Springer Nature. (**d**) Band alignment of the CZTSSe/CdS heterojunction and the corresponding J-V characteristics of the solar cells following a two-step annealing treatment. Adapted with permission from [117]. Copyright © 2021, American Chemical Society. (**e**) Comparison of device efficiency for two-step deposition annealing, one-step deposition annealing, and non-post-deposition annealing processes. Adapted with permission from [118]. Copyright © 2019, Elsevier.

Heterojunction heat treatment (JHT) improves the quality of the interface between the CdS buffer and the CZTSSe absorber layer by controlling the annealing temperature and time. The aim of this process is to reduce interfacial defect states, optimize band alignment and promote element diffusion to enhance carrier transport. JHT has been extensively used in high-efficiency CZTSSe solar cells, and the mechanisms have been proposed to explain its performance [112]. In 2014, Mitzi et al. optimized the annealing conditions for the CdS/In_2_S_3_ double-emitter structure using the rapid thermal annealing (RTA) technique. They discovered that this treatment enabled the controlled diffusion of the In element into the CdS buffer layer and the CZTSSe absorber layer, ultimately reducing the V_oc_ deficit to below 600 mV and the highest PCE of the device reached 12.7% [113]. In 2018, the team of Hao used a heat treatment at 300 °C to the CZTS/CdS heterojunction, effectively promoting the interdiffusion of elements at the interface and directly inducing Cd atoms to occupy the Zn/Cu lattice sites, thereby leading to local Cu deficiency in the Na-accumulated region of heterojunction. As a result, new ultrathin layers of Cu_2_Cd_x_Zn_1-x_SnS_4_ and Zn_x_Cd_1-x_SnS_4_ at the interface were formed, with a better band alignment and significantly reducing non-radiative recombination in the heterojunction region (Figure 13a). This strategy enabled the PCE of pure sulfide-based CZTS cells to reach 11% [114]. Later, to solve the V_oc_ and FF deficits which were due to the presence of the antisite defects and the poor heterojunction interfaces, the team further investigated the mechanism of the heterojunction annealing based on the Cd-alloyed CZTS (CZCTS) absorber layer. This time, they adopted the CZCTS/CdS/ITO structure and subjected it to a 300 °C annealing treatment. The findings indicated that this process not only improved the conductivity and transmittance of the ITO layer, but also improved moderate element interdiffusion of Cd, Zn, In, and Sn. This elemental diffusion increased the electron and hole densities near the depletion region and suppressed the interfacial defect density, ultimately enabling the pure sulfide CZTS solar cell to achieve an efficiency breakthrough of 12.6% [115]. Although high-temperature annealing effectively promotes elemental interdiffusion, excessive temperatures can lead to uncontrolled diffusion. Consequently, exploring interface regulation under moderate and low temperatures has become a new research direction.

Through low-temperature sufficient annealing in the deposition process of Al-doped ZnO films, Guo et al. designed and successfully constructed the bandgap gradient structure in CZTSSe thin films (Figure 13b). This annealing process promoted the mutual diffusion of Cu^+^ and Cd^2+^ elements at the heterojunction interface, causing the Cd^2+^ to be gradient-distributed in CZTSSe, and partially converting n-type CdS to p-type Cu_2_S, forming a bandgap gradient structure of CZTSSe:Cd_low_/CZTSSe:Cd_high_/Cu_2_S/CdS. The band alignment between the p–n junction not only improves the electron transport but also reduces carrier recombination. As a consequence, the V_oc_ and FF are significantly improved, and a certified PCE of 12.25% is obtained. Compared to direct high-temperature annealing, this low-temperature process more effectively suppressed CdS grain agglomeration while regulating elemental interdiffusion to form a beneficial interface [116]. Gong et al. used a low-temperature heat treatment on the ACZTSSe/CdS heterojunction. Through characterization, it was found that the improvement in device performance was mainly attributed to the reduction in the defect concentration at the heterojunction interface. This low-temperature heat treatment caused elemental redistribution and diffusion at the heterojunction, driving migration of Cd^2+^ from the absorber back to CdS and migrating Zn^2+^ from the absorber bulk to the surface. Eventually, a gradient distribution of Zn and Cd elements near the interface was achieved, and the formation of the ACZTSSe/CdS epitaxial interface was promoted (Figure 13c). This resulted in a 13.0% NREL certified device efficiency for ACZTSSe solar cells [40]. Duan et al. proposed a two-step heterogeneous junction annealing strategy, including a low-temperature process at 90 °C under a N_2_ atmosphere for 1 h, and a high-temperature process at 210 °C in air for 1 min. This method optimizes the band structure while maintaining the crystallinity and coverage of the CdS layer, and achieves a heterojunction band alignment with a smaller “spike” barrier. It reduces the CBO from 0.39 eV to 0.24 eV, thereby minimizing the interfacial recombination loss. Moreover, the approach mitigates the shortcomings of the high-temperature direct treatment, including more pinholes on CdS films and unnecessary diffusion of elements. This resulted in a PCE of 12.3% for the CZTSSe solar cells (Figure 13d) [117]. Meng et al. developed a new two-step deposition strategy. This process involved pre-depositing a thin CdS layer on the CZTSSe absorber via CBD, followed by a mild heat treatment, and subsequently covering the surface with a thicker CdS layer. The initially heat-treated CdS layer facilitated beneficial ion interdiffusion and optimized the band alignment. The subsequently deposited CdS layer filled the voids generated during the annealing process, reducing shunt losses [118]. In comparison with the devices that have the conventional CBD-deposited CdS layer without and with the annealing process, the two-step technique dramatically increased the V_oc_, J_sc_ and FF of the device, as well as the PCE (Figure 13e) [118]. In 2025, Zhang et al. discovered that a lower pre-annealing temperature significantly improves the microstructure of the precursor. This optimization not only facilitates selenium penetration but also promotes grain growth through Ag during the selenization process, thereby enhancing the overall quality of the absorber layer [41]. Consequently, the carrier separation and transport efficiency at grain boundaries are significantly enhanced, while both bulk and interfacial defects were reduced, effectively suppressing carrier recombination. Ultimately, a flexible CZTSSe solar cell with a certified efficiency of 13.22% was achieved. This work elucidates the critical impact of pre-annealing on crystal growth and proposes an effective strategy for enhancing the quality of kesterite films [41].

In summary, as a simple and effective strategy, JHT has become a method for obtaining highly efficient solar cells. By precisely controlling the annealing temperature and time, combined with methods such as cation engineering, buffer layer design and interface modification, researchers have achieved the directional regulation of element diffusion, significantly reducing interface defect states and optimizing the band alignment. These advancements have propelled the efficiency of CZTSSe solar cells, laying a solid foundation for their industrial application.

#### 3.2.4. Optimizing Heterojunction Interface Performance via Passivation Layers

The function of interfacial passivation layers in CZTSSe solar cells represents a research area of considerable interest, primarily due to their ability to effectively mitigate non-radiative recombination at the CZTSSe/CdS interface. Studies indicate that introducing an ultrathin intermediate layer between the absorber and the buffer layer is a highly effective passivation strategy. To date, researchers have developed various materials as passivation layers for CZTSSe, including TiO_2_, SnO_2_, Al_2_O_3_, and In_2_S_3_. These materials function by repairing interfacial defects, optimizing band alignment, and improving physical contact, thereby suppressing interfacial recombination and enhancing the performance of CZTSSe cells.

**Figure 14:**
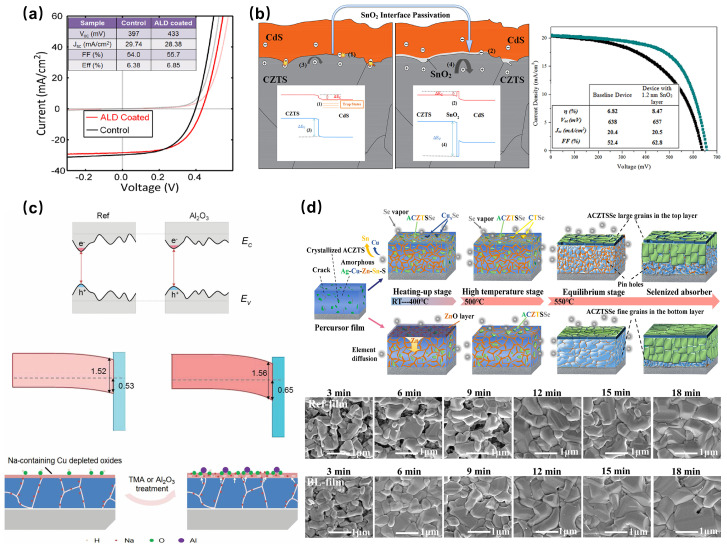
(**a**) J-V characteristics for the CZTSSe control device and the ALD coated device. Adapted with permission from [119]. Copyright © 2014, AIP Publishing. (**b**) Schematic energy band diagram of the CZTSSe/SnO_2_/CdS heterojunction and the corresponding J-V curves. Adapted with permission from [120]. Copyright © 2018, American Chemical Society. (**c**) Schematic illustration of electronic band edge fluctuations in CZTS materials with and without Al_2_O_3_ treatment, alongside the proposed mechanism for surface modification of the CZTS absorber induced by the ALD-Al_2_O_3_ process. Adapted with permission from [123]. Copyright © 2019, Royal Society of Chemistry. (**d**) Schematic diagram of the selenization process, accompanied by top-view SEM images illustrating the morphological evolution of reference films (R-films) and blocking layer films (BL-films) after selenization for 3, 6, 9, 12, 15, and 18 min, respectively. Adapted with permission from [130]. Copyright © 2025, Royal Society of Chemistry.

Wu et al. employed ALD to deposit a 1–2 nm TiO_2_ film on the CZTSSe surface, which successfully reduced interfacial recombination and improved device performance, as shown in Figure 14a. Characterization via I-V curves, impedance spectroscopy and CV/DLCP revealed that the TiO_2_ passivation layer increased the V_oc_ from 397 mV to 433 mV. This enhancement was attributed to a 42 meV increase in the activation energy of the recombination process and a reduction in interface defect density, confirming that TiO_2_ effectively suppresses interfacial recombination and offers a viable solution for improving the efficiency of low-cost CZTSSe devices [119]. Sun et al. adopted the successive ionic layer adsorption and reaction (SILAR) method to deposit an ultrathin SnO_2_ intermediate layer for defect passivation and band alignment optimization at the CZTS/CdS interface (Figure 14b). Following the introduction of a 1.2 nm SnO_2_ layer, the CZTS cell efficiency rose from 6.82% to 8.47%, with a V_oc_ of 657 mV and an FF of 62.8%. This work provides a new insight into the passivation of CdS/CZTS interface, suppression of shunt pathways and optimization of band alignments, based on solution-processed SnO_2_ [120]. Lee et al. demonstrated the efficacy of inserting a nanometer-scale Al_2_O_3_ layer between the CZTS absorber layer and the CdS buffer layer. Their research showed that this inclusion not only effectively passivated interfacial defects, improving V_oc_ and FF, but also enhanced long-wavelength collection efficiency and J_sc_, ultimately increasing the net efficiency of the device by approximately 15% [121]. However, Park et al. offered a different opinion, arguing that Al_2_O_3_ does not directly passivate defects at the CZTS/CdS interface because the NH_4_OH present during CdS deposition can etch away the Al_2_O_3_ surface. Instead, they attributed the performance enhancement to hydrogen gas generated near the CZTS/Al_2_O_3_ interface during the ALD process. These hydrogen molecules play a passivation role by passivating dangling bonds on the CZTS surface [122]. In a Cd-free CZTS/ZnSnO system, Cui et al. found that a full ALD-Al_2_O_3_ cycle and trimethylaluminum (TMA) exposure treatment could significantly enhance V_oc_, linking this to the properties of the CZTS interface. Both processes facilitate the formation of a thicker Cu-deficient nanolayer with enriched Na and O on the CZTS surface. This nanolayer acts as a uniform passivation layer that reduces local band edge fluctuations, widens the electronic bandgap and suppresses defect-assisted recombination at the heterojunction interface, thereby improving the V_oc_ and FF (Figure 14c). Due to the ability of the nanolayer to alter the atomic composition of the surface area of the material, it becomes a universal method for surface passivation [123]. Building on this, Xie et al. developed a facile, rapid method using AlCl_3_ and CH_3_CSNH_2_ aqueous solutions to insert an Al(OH)_3_ nanolayer at the CdS/CZTSSe interface. This passivation effectively inhibited interfacial recombination and substantially increased shunt resistance, collectively enhancing the V_oc_ and FF to achieve an efficiency of 9.1% [124].

Being an indirect bandgap semiconductor material, In_2_S_3_ has higher transparency than CdS, and its bandgap is 2.1 eV [125,126]. Such characteristics enable it to be used as a buffer layer material for CZTSSe devices, which is expected to reduce the light absorption loss of the buffer layer, reduce the band mismatch and interfacial recombination between the buffer layer and CZTSSe, and improve J_sc_. Barkhous et al. reported that the CBO between In_2_S_3_ and the CZTSSe absorber layer with a specific composition (S/(S + Se) = 0.4) was 0.15 ± 0.1 eV, which belongs to a “spike-like” band alignment within the optimal range. Benefiting from the above advantages, the CZTSSe solar cells adopting a single In_2_S_3_ buffer layer achieved a PCE of 7.6%, a performance comparable to that of the control group using a CdS buffer layer [125]. Therefore, the In_2_S_3_ modification layer introduced at CZTSSe/CdS interface has been examined to improve front interface roughness and eliminate interfacial recombination [127]. Yan et al. developed CdS/In_2_S_3_ mixed buffer layer. They placed In_2_S_3_ between CdS and CZTS, and then carried out rapid thermal processing (RTP) annealing. This produced a V_oc_ over 710 mV and increased J_sc_ from 15.9 mA/cm^2^ to 17.6 mA/cm^2^. Eventually, the efficiency was improved from 5.47% to 6.62% [128]. Song et al. established that thin In_2_S_3_ was deposited on the CZTSSe surface to not only successfully reduce non-radiative recombination through a reduction in deep-level defects and increasing minority carrier lifetime, but also provide a physical defect passivation method. This modified layer can fill voids on the CZTSSe surface, forming a more regular interface, promoting the uniform growth of the next layer CdS and avoiding agglomeration. This strategy increased the cell efficiency from 7.33% to 9.24%, which not only provides an effective solution to boost the single-junction CZTSSe type of cell, but also lays the foundation for its application in tandem solar cells [129]. To address the issues of deep-level defects and V_oc_ deficit due to complex phase evolution path in CZTSSe solar cells, Chi et al. proposed a strategy of sputtering ZnO thin film over the precursor surface to act as a sacrificial layer. By taking advantage of the stable property of the ZnO layer at low temperatures, the contact and reaction between the CZTS precursor and the low-concentration Se are effectively blocked, thereby inhibiting the complex intermediate phase evolution. As the temperature rises, the ZnO layer gradually spreads and eventually disappears, prompting the precursor to undergo a one-step phase transformation with high-concentration Se at high temperatures, directly converting into the CZTSSe phase, as shown in Figure 14d. Compared with the unmodified sample (R film), in which phase-transition nucleation during the low-temperature stage results in the formation of a significant number of relatively small crystal nuclei due to the limited driving force, the modified sample exhibits improved crystal morphology with larger grains and a denser structure. Eventually, this high crystalline quality and low defect density device achieves a PCE of 14.45%, a V_oc_ of 572.6 mV, and a low V_oc_ deficit (V_oc_/$V_{\mathrm{OC}}^{\mathrm{SQ}}$ = 69.7%), providing further breakthrough possibilities for the performance optimization of kesterite solar cells [130].

## 4. Challenges and Future Prospects

As discussed above, interfacial recombination is the major cause of the V_oc_ deficit in CZTSSe solar cells, as it greatly limits the improvement of device PCE. To solve this issue, researchers have employed various methods in an attempt to overcome the problem of V_oc_ deficit. Despite the use of various interface engineering methods such as introduction of intermediate layers, alkali metal post-deposition treatments and heterojunction heat treatments to control this problem, the key challenge remains in achieving atomic-level, precise, controllable, and stable regulation of the surface chemical state and electronic structure of the CZTSSe absorber layer. Although interface engineering has been proven to be an effective method of solving such problems, there are still many obstacles to its implementation in practical applications. Based on the current research progress, future studies should focus on the following directions:

(1) Back Interface Optimization Strategies

Interfacial decomposition reactions and high recombination densities at the back interface are important bottlenecks that limit performance of the device. The future research should focus on two core directions: on the one hand, developing barrier layers with excellent chemical stability to effectively suppress side reactions at the back interface; on the other hand, designing multifunctional intermediate layers that are both chemically inert and electrically conductive. For instance, two-dimensional (2D) materials such as graphene [131] and MXene can be employed as barrier layers to suppress excessive reactions between the Mo back contact and the S/Se atmosphere, thus eliminating the formation of thick Mo(S,Se)_2_ layers.

(2) Heterojunction Interface Optimization

Constructing an optimal band alignment, ideally with a “spike-like” structure, is a key process for enhancing the performance of CZTSSe photovoltaic devices. For example, by utilizing a graded-bandgap CdS buffer layer and adjusting its composition and processing, the CBM at the heterojunction interface can be precisely tuned [132,133,134]. This precise control will prevent the severe interfacial majority carrier recombination associated with “cliff-like” structures, while simultaneously mitigating insufficient band bending at the interface. Additionally, future research should shift its focus away from traditional CdS buffer layers toward the development of novel, environmentally friendly and wide-bandgap alternative materials [135].

(3) Tandem Solar Cells

In the pursuit of efficient photovoltaic technologies, CZTSSe has become a promising material in tandem solar cells due to its advantages such as tunable bandgap (1.0–1.5 eV), abundant reserves of constituent elements, eco-friendliness and low cost [136,137,138]. Current research shows that CZTSSe can be used in perovskite, CIGS and silicon-based cells to construct various tandem structures [139,140,141], offering diverse technical paths to surpass the efficiency limits of single-junction cells. The potential of this technological path has been demonstrated by the relevant experiments.

In the perovskite/CZTSSe solar cell, Sun et al. achieved efficient monolithic two-terminal perovskite/CZTSSe tandem solar cells through interface optimization technology, and the best performing tandem device exhibited a high conversion efficiency of 17.5% without the hysteresis effect [142]. A perovskite/CZTSSe four-terminal (4-T) tandem device with an efficiency of 22.27% was achieved by Xu et al. through combining wide-bandgap perovskite devices with narrow-bandgap CZTSSe [143]. In addition, Patil et al. explored the introduction of diphenylammonium chloride (DPACl) as an additive into the wide-bandgap perovskite layer to enhance the performance of 4-T hybrid tandem solar cells, further raising the PCE to 22.96% [144]. Apart from the perovskite system, CZTSSe is also applicable to other stack structures. Theoretical simulations have indicated that CZTS/Si tandem cells can reach a PCE of 23% [145]. Moreover, the TCAD Silvaco-simulation is used to optimize the combination of CZTSSe and CIGS device structure. The new bottom sub-cell CZTSSe/CIGS structure has broken the Shockley–Queisser limit, with an efficiency of over 35% and a V_oc_ of approximately 1.7 V [146]. These studies highlight the enormous application potential of CZTSSe in various tandem architectures and this offers insights into the production of high-efficiency and low-cost photovoltaic devices.

## 5. Conclusions

This review summarizes the development process of CZTSSe thin-film solar cells, with a focus on the current interface-related issues in this field and the latest research progress in interface engineering. It elucidates the key mechanisms that lead to the performance bottleneck of the device, especially the excessive growth of Mo(S,Se)_2_ at the back interface, the formation of voids and secondary phases, as well as the severe V_oc_ deficit caused by the band structure mismatch and deep-level defects at the front interface. Research shows that interface engineering as a core regulatory strategy, can effectively optimize the interface band alignment, passivate interfacial defects, and suppress non-radiative recombination, thereby enabling CZTSSe solar cells to demonstrate unique competitive advantages in the emerging field of photovoltaic technology.

## Data Availability

No new data were created or analyzed in this study. Data sharing is not applicable to this article.
